# *In Vitro* Evaluation of the Biological Availability of Hyaluronic Acid Polyethylene Glycols-Cross-Linked Hydrogels to Bovine Testes Hyaluronidase

**DOI:** 10.1155/2019/3196723

**Published:** 2019-06-12

**Authors:** N. Zerbinati, R. Mocchi, H. Galadari, C. Maccario, M. Maggi, R. Rauso, A. Passi, C. Esposito, S. Sommatis

**Affiliations:** ^1^Università degli Studi dell'Insubria Department of Medicine and Surgery, Varese, Italy; ^2^UB-CARE S.r.l. Spin-Off University of Pavia, Pavia, Italy; ^3^Department of Medicine, College of Medicine and Health Sciences, UAE University, Al Ain, UAE; ^4^Università degli Studi di Pavia, Department of Molecular Medicine, Pavia, Italy; ^5^Università degli Studi della Campania “Luigi Vanvitelli”, Department of Maxillofacial Surgery, Napoli, Italy; ^6^Centro Medico Polispecialistico, Pavia, Italy

## Abstract

During last years, hyaluronic acid- (HA-) based dermal fillers have grown rapidly and continuously, as reported by the American Society of Aesthetic Plastic Surgery (ASAPS). In fact, HA fillers are considered the gold standard technique for soft tissue augmentation, deep skin hydration, and facial recontouring, playing a key role as an alternative to plastic surgery. HA fillers are less invasive, more biocompatible, and safer and with a more natural and immediate result if compared to plastic surgery. Hence, the safety of HA-based dermal fillers plays a crucial role, mostly in terms of biocompatibility and adjustability in case of unpleasant results and side effects such as, tyndall effect, edema, or granulomas. Hyaluronidase is a naturally occurring enzyme, present in the human body, and can degrade HA fillers avoiding more severe complications. In this article, we analyzed the bioavailability of hyaluronidase degradation of five fillers of Neauvia® hydrogels line (MatexLab SA, Lugano, CH), composed of pure hyaluronic acid and based on PEGDE cross-linking (polyethylene glycol) technology that guarantees a higher biocompatibility and an optimal biointegration and rheological characteristics. The performed* in vitro* testing is based on the colorimetric determination of the N-acetyl-D-glucosamine (NAG) present in solution after incubation with hyaluronidase, determined at different time points in order to assess the kinetic of each product degradation (1h, 3h, 6h, 24h, 48h, 72h, 120h, and 168h). The aim of this study was to assess,* in vitro,* how the difference in HA content and PEGDE concentration of the analyzed fillers can influence the product biocompatibility, intended as product enzymatic clearance and duration in time. The results demonstrated that the method was reproducible and easy to perform and that all the analyzed fillers are naturally immediately available for hyaluronidase-mediated degradation.

## 1. Introduction

During last years, the demand for ever less invasive but equally effective techniques in the field of aesthetic medicine has increased exponentially. This trend has therefore stimulated the use of intradermal fillers as a gold standard method to increase soft tissue volume, at the expense of plastic surgery, a technique considered much more invasive [1–4]. In fact, if compared to an implant device, intradermal fillers are safer and less invasive, with an immediate result and a more natural effect [2].

As public awareness and acceptance of dermal fillers grows, the size of market grows also with an estimation of 160 products currently available worldwide on the market and produced by more than 50 companies [5]. Moreover, according to data from the American Society for Aesthetic Plastic Surgery (ASAPS), more than 1.6 million dermal filler treatments were performed in 2011, making them the second most popular nonsurgical cosmetic procedure performed in the USA after neuromodulators, whose procedure is frequently performed concurrently with dermal filler injections [6].

Moreover, according to the ASAPS, more than 85% of all dermal filler procedures performed in 2008 used hyaluronic acid- (HA-) based products [2] and in 2015, HA-dermal fillers accounted for more than 92% of all filler treatments in the US [7].

The peculiarities of HA, such as the ability to increase the volume of skin being highly biocompatible, its nonimmunogenic nature, to encompass a large volume of water that expands extracellular space and to hydrate tissues, make it the first choice as the major component of dermal fillers [8, 9].

It is important to underline that HA is physiologically present in the human body, being a major component of the extracellular matrix [10], playing a major role in its organization and integrity, thereby participating in the preservation of the form and in the spatial arrangement of tissue components.

HA is a glycosaminoglycan (GAG) polymer consisting of repeated disaccharide units of glucuronic acid and N-acetylglucosamine, which are linked by *β*(1,4)-glycosidic bond, reaching 10^5^-10^7^ Da in molecular mass [11–13].

Even if HA-based fillers are considered safe and noninvasive, side effects can occur; in fact, unwanted some adverse reactions have been reported such as overcorrections, tyndall effect, lower eyelid edema to granulomas, infections, or even vascular occlusion [14].

Therefore, the presence of a specific antidote, e.g., hyaluronidase, becomes important for the management of complications during and after filler injection. Hyaluronidases are endoglycosidases, physiologically present in the human body, that cleave HA, reducing its viscosity [15]. Hyaluronidases could be classified into three groups according to their mechanism of action and end products: mammalian hyaluronidase (testis tube), leech/hook worm hyaluronidase, and microbial hyaluronidase [16, 17]. Mammalian and microbial hyaluronidases act on the *β*-1,4-glycosidic linkages of HA, while the leech/hook worm hyaluronidase degrades the *β*-1,3-glycosidic bond; all of them degrade the HA molecule and produce oligosaccharides of different chain lengths [18, 19].

Hyaluronidase is able to degrade HA-hydrogels and may rescue from more severe vascular complications. For this reason, immediate availability of hyaluronidase is essential for every physician who uses HA fillers [20–22]. Moreover, the sensitivity to endogenous hyaluronidases produced by the body is an important factor to be considered for the temporal persistence of the filler once injected. In fact, this mechanism plays a fundamental role in achieving a short- or a long-lasting effect of the product.

Despite the availability of hyaluronidases, it has being discussed whether all HA fillers are sensitive to the activity of these enzymes; in fact, several studies have shown that the resistance to degradation depends on numerous factors, such as the concentration of HA, the type and degree of cross-linking, and the cohesive properties [23–26].

Considering the potential risk related to HA filler injection, it is extremely important to analyze and deeper investigate the sensitivity of commercially available HA-based fillers to hyaluronidase degradation in order to guarantee a greater safety to the patients and to offer a potential competitive edge over other manufacturers of dermal fillers.

**Figure 1:**
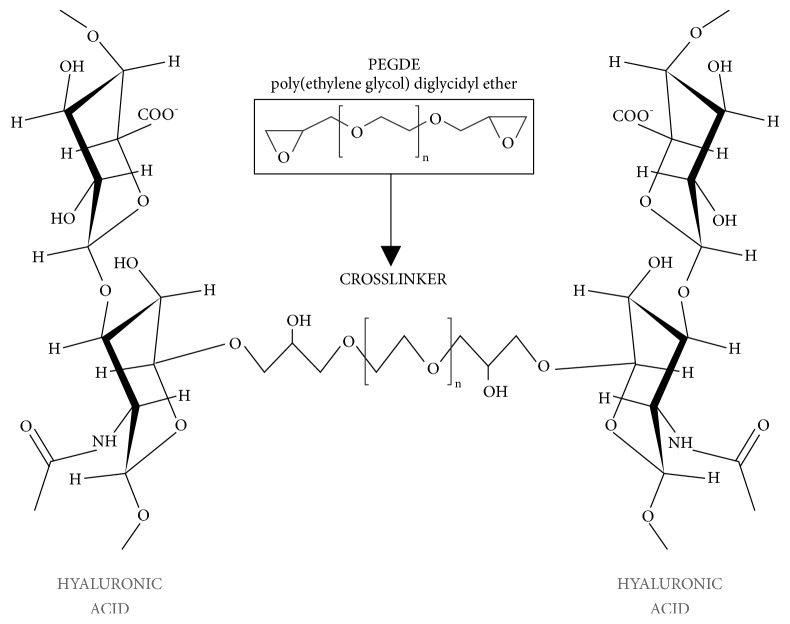
PEGDE structure and crosslinking with two molecules of hyaluronic acid.

In order to assess these important aspects, we analyzed the bioavailability of bovine hyaluronidase degradation of five fillers of Neauvia® hydrogels line (MatexLab SA, Lugano, CH) in a time-dependent manner [27]. The Neauvia® hydrogels are composed of pure hyaluronic acid and are based on PEGDE cross-linking (polyethylene glycol) (Figure 1) technology [28–30] that guarantees a major biocompatibility and an optimal biointegration and rheological characteristics.

Each Neauvia® filler that we tested is based on a specific combination of HA and PEGDE for different treatment indications and injection plans, according to each product's characteristics.

## 2. Methods

### 2.1. Chemicals and Instruments

The bioavailability of hyaluronidase of five Neauvia® hydrogels has been evaluated* in vitro*. These products are composed of pure hyaluronic acid and a PEGDE cross-linker that can guarantee a high level of biosafety and tolerability profile, as well as a 3D hydrogel matrix.

These hydrogels differ in hyaluronic acid content and PEGDE concentration and the different specific combination allows creating products with different indications and injection plans and with a good rheological ratio.

Type I-S hyaluronidase from bovine testis was purchased from Sigma Aldrich (ref. H3506, 451 Units/mg); all other chemicals were of the highest purity available.

The absorbance was measured using a Multiskan-Go (Fisher Scientific) spectrophotometer.

### 2.2. Sample Preparation

All the Neauvia® hydrogels were weighed (0.2 g) and placed in glass tubes. The tubes were then centrifuged for 5 minutes at 1000 g using a refrigerated bench centrifuge (Megastar 600R, VWR) equipped with a swinging bucket rotor. At the end of the centrifugation, thin pellets firmly attached at the bottom of the tubes were obtained.

### 2.3. Hyaluronidase Sensitivity Test

Type I-S hyaluronidase from bovine testes (Sigma Aldrich; ref. H3506, 451 U/mg) was prepared at 6080 U/ml in isotonic phosphate-NaCl (0.9%) buffer at pH 7.4 [31]. The glass tubes containing the gel pellets and the hyaluronidase solution were preincubated separately at 37°C. Then, 100*μ*l of the enzyme solution was added gently onto the surface of the gels and, after incubation at different time points (1h, 3h, 6h, 24h, 48h, 72h, 120h, 168h), the enzymatic reaction was stopped by the addition of 0.1 ml potassium tetraborate (0.8 M, pH 9.1), followed by vortexing and heating at 100°C. The tubes were then cooled at room temperature and the NAG present in solution was assayed.

### 2.4. Assay of the Released NAG

The measurement of the NAG present in solution was performed according to the methods reported in Sall et al. and Reissing et al. [23, 31]. Briefly, Ehrlich's reagent (Sigma Aldrich) diluted 1:10 in acetic acid was added to the tubes. The samples were vortexed and incubated for 20 min at 37°C, to develop a violet color proportional to the NAG content in each sample. The tubes were centrifuged at 1000g for 15min to remove gel fragments and protein precipitate. Then, each sample absorbance at 585nm was recorded using a microplate reader (Multiskan-Go, Fisher Scientific). A blank condition consisting only of phosphate buffer and the Ehrlich's reagent was set up for each reaction.

### 2.5. Data Analysis

Data obtained from hyaluronidase sensitivity tests were analyzed by determining NAG degradation percentage at each time point. The expected amount of NAG in each sample starting from the percentage of hyaluronic acid in each product was calculated. The obtained values were used as a reference to calculate the percentage of NAG present in solution after hyaluronidase-mediated degradation.

The obtained data were plotted using the standard hyperbole equation (Sigma plot)

(1) $$\begin{array}{c} y=\frac{ax}{b+x} \end{array}$$

a = degradation maximum; b = t(1/2).

Data points fitting to the model were evaluated by calculating for each sample analysis R^2^.

Slope values between points 0 - 50% were calculated as well in order to determine the degradation rate for each product. t(1/2) is defined as the time at which NAG degradation is half the maximum;

t_50%_ is defined as the time at which NAG degradation is equal to 50%.

## 3. Results

**Figure 2:**
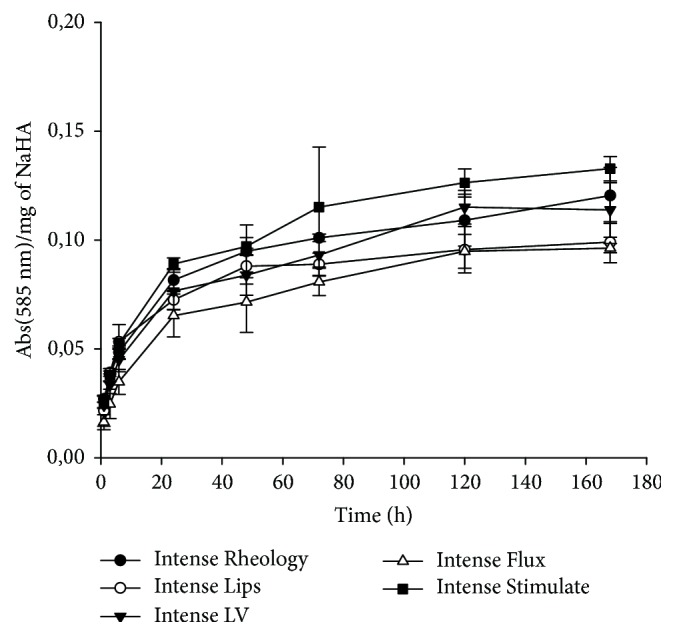
Hydrogels sensitivity to hyaluronidase. Absorbance values obtained after colorimetric NAG assay. Values are normalized for sample content in HA.

All the tested hydrogels resulted to be sensitive to hyaluronidase degradation. Indeed, the incubation of each of the analyzed samples in the presence of bovine hyaluronidase resulted in a time-dependent change of absorbance at 585 nm (Figure 2).

According to the coefficients of determination reported in Table 1, hyaluronidase degradation curves resulted within the hyperbola regression in all of the cases indicating that, independently from the product HA content and cross-linker concentration, all five analyzed products are naturally immediately available for enzyme-mediated degradation.

**Figure 3:**
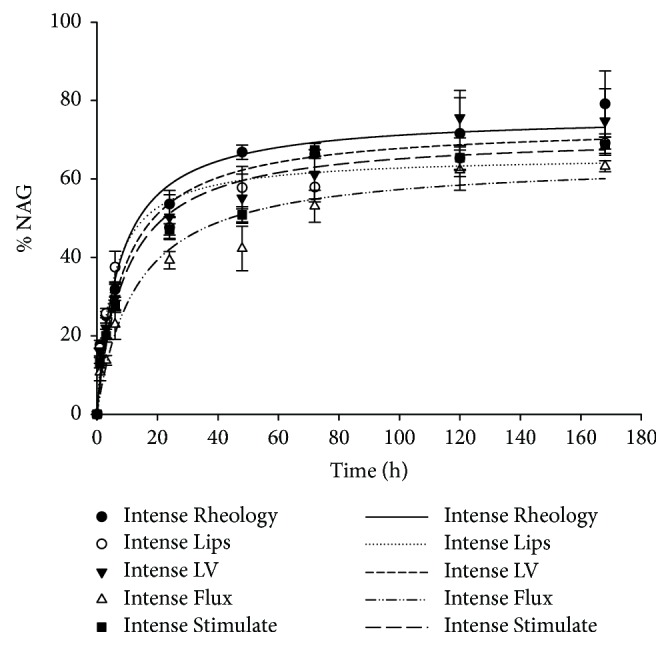
Percentage of NAG released after hyaluronidase degradation assay. Data were plotted using the hyperbola equation.

The maximal percentage of NAG degradation (76.36 %) was obtained with the product Neauvia® Intense Rheology containing 22 mg/ml HA. Similar degradation maxima were obtained for products Neauvia® Intense LV (26 mg/ml HA, degradation max 73.86%) and Neauvia® Stimulate (26 mg/ml HA containing CaHA, degradation max 71.24%). Degradation maxima were lower for products Neauvia® Intense Lips (24 mg/ml HA) and Neauvia® Flux (26 mg/ml HA), 65.95% and 64.7%, respectively (Figure 3). As for the degradation rate, evaluated by calculating each curve slope between 0 and 50%, Neauvia® Intense Lips resulted to have the quickest degradation rate (6.54 %/h), while Neauvia® Flux resulted to have the slowest degradation rate (2.5 %/h). Indeed, Neauvia® Intense Lips t_1/2_ resulted to be the highest (5.04 h) while Neauvia® Flux the lowest (12.9 h).

## 4. Discussion

In this study, the bioavailability of hyaluronidase degradation of five Neauvia® hydrogels has been evaluated* in vitro*. The five tested products differ for hyaluronic acid content and PEGDE cross-linker concentration. The aim of this study was to assess,* in vitro,* how the previously mentioned features of a filler can influence the product biocompatibility, intended as product enzymatic clearance, and consequently the duration over time of the implant. In particular, the use of PEGDE as a crosslinking agent is an innovation in the biomedical field and makes interesting the study of Neauvia® PEGDE fillers in order to investigate their behavior in the presence of hyaluronidase mostly in terms of improved stability and preserved biocompatibility. Indeed, ideally a filler should be endowed with long term stability but, at the same time, with plasticity and should be biocompatible. Here, by using an* in vitro* “stress” assay, we determined the sensitivity of Neauvia® Intense Rheology, Neauvia® Intense Lips, Neauvia® Intense LV, Neauvia® Flux, and Neauvia® Stimulate to a bovine hyaluronidase. The results obtained showed that all the tested products were sensitive to hyaluronidase degradation, but the maximum degradation percentage obtained in the experimental conditions was not dependent on HA initial concentration nor on cross-linker concentration. Similar behavior was found when considering the calculated degradation rates. Indeed Neauvia® Intense Lips (24 mg/ml HA) was degraded faster and Neauvia® Flux (26 mg/ml HA) was degraded slower. Our result demonstrated that HA content can be correlated instead to t_50%_, indicating that HA content was related to initial hydrogel degradation.

In conclusion, we found a correlation between HA content and short-term degradation, instead long-term degradation of the analyzed hydrogels is very likely influenced by different biophysics characteristics of the hydrogels.

## Figures and Tables

**Table 1:** Neauvia® hydrogels characteristics and degradation parameters.

Product	HA content	Crosslinker	R^2^	Deg max	t_1/2_	t_50%_	Slope
Neauvia® Intense Rheology	22 mg/ml	PEGDE	0.95	**76.36**%	7.32 h	**13.9 h**	5.22%/h
Neauvia® Intense Lips	24 mg/ml	PEGDE	0.97	65.95%	**5.04 h**	15.8 h	**6.54**%**/h**
Neauvia® Intense LV	26 mg/ml	PEGDE	0.94	73.86%	8.95 h	18.75 h	4.13%/h
Neauvia® Flux	26 mg/ml	PEGDE	0.96	64.70%	12.90 h	43.88 h	2.50%/h
Neauvia® Stimulate	26 mg/ml	PEGDE	0.97	71.24%	9.39 h	22.10 h	3.79%/h

Best values for each parameter are highlighted in bold.

## Data Availability

The processed data used to support the finding of this study are included within the article. The raw data used to support the finding of this study are available from the corresponding author upon request.
